# Upregulation of CCL7, CCL20, CXCL2, IL-1β, IL-6 and MMP-9 in Skin Samples of PCB Exposed Individuals—A Preliminary Study

**DOI:** 10.3390/ijerph18189711

**Published:** 2021-09-15

**Authors:** Marike Leijs, Katharina Fietkau, Hans F. Merk, Thomas Schettgen, Thomas Kraus, André Esser

**Affiliations:** 1 Department of Dermatology and Allergology, Medical Faculty, RWTH Aachen University, 52072 Aachen, Germany; kfietkau@ukaachen.de (K.F.); Hans.Merk@post.rwth-aachen.de (H.F.M.); 2 Department of Dermatology, St. Nikolaus Hospital Eupen, 4700 Eupen, Belgium; 3 Institute for Occupational, Social and Environmental Medicine, Medical Faculty, RWTH Aachen University, 52072 Aachen, Germany; tschettgen@ukaachen.de (T.S.); tkraus@ukaachen.de (T.K.); anesser@ukaachen.de (A.E.)

**Keywords:** polychlorinated biphenyls, gene regulation, HELPcB, PCB exposure, CCL20, CXCL2, IL-6, IL-1β, MMP-9, CCL-7, EPGN, NRF2

## Abstract

Polychlorinated biphenyls (PCBs) are well known immunotoxic and carcinogenic compounds. Although cutaneous symptoms are the hallmark of exposure to these compounds, exact pathophysiologic mechanisms are not well understood. We took skin biopsies from moderately high PCB exposed workers (*n* = 25) after an informed consent and investigated the expression of immunological markers such as CCL-7, CCL-20, CXCL2, IL-1β and IL-6, as well as the matrix metalloproteinase MMP-9, EPGN and NRF2 by RT-qPCR, and compared expression levels with plasma PCB levels. Statistical analyses showed a significant correlation between CCL-20, CXCL2, IL-6, IL-1β, CCL-7 and MMP-9 and PCB serum levels. EPGN and NRF2 were not correlated to PCB levels in the blood. We found a significant correlation of genes involved in autoimmune, auto-inflammatory and carcinogenesis in skin samples of PCB exposed individuals with elevated plasma PCB levels. Confirmation of these findings needs to be performed in bigger study groups and larger gen-sets, including multiple housekeeping genes. Further study needs to be performed to see whether a chronical exposure to these and similar compounds can cause higher incidence of malignancies and inflammatory disease.

## 1. Introduction

Polychlorinated biphenyls (PCBs) are bioaccumulative and toxic environmental pollutants that elicit a broad spectrum of negative health effects including endocrine disruption as well as immunotoxic effects [1,2,3]. This group of 209 congeners were, since 1930 till their ban in 1977, used in large amounts for their fire resistant and low electrical conductivity [4]. After ongoing studies, PCBs have been classified as human carcinogens group 1 by the International Agency for Research on Cancer (IARC) since 2013 [5,6]. Humans are exposed via ingestion (food, drinking water), via inhalation, and via dermal contact. Due to long half-lives [7] exposure to PCBs still occurs in daily live and background levels of these compounds have been found throughout the industrialized world. Levels mostly depending on age, dietary habits and place of residence [8,9]. While individual congeners differ markedly in their chemical and toxicological properties, mainly depending on the position of the chlorine atoms on the PCB molecule, certain classes of PCB congeners have common mechanisms of action with regard to their toxicity [10]. Twelve congeners with co-planar structure show toxicological properties similar to dioxin and are therefore termed dioxin-like PCBs (DL-PCBs).

Cutaneous symptoms are the hallmark and most obvious signs of exposure to these compounds [11]. As an example, during the Yusho incident (Japan, 1968), where humans were exposed to PCB contaminated oil, a disease characterized by acne-like eruptions, pigmentation of the skin, and eye discharge appeared. In total, more than 1800 patients have been registered as having the “Yusho disease” and around 300 are deceased [12,13]. In addition, a biodegradative function in the metabolism of dioxin-like compounds have been shown in one other study [14]. The precise pathophysiology of cutaneous effects such as chloracne has not been identified. It has been postulated that multiple reactions related to the cell proliferation are responsible. An induction of p-EGFR (epidermal growth factor receptor), p-MAPK (mitogen-activated protein kinase) and CK17 (cytokeratin-17) mRNA and protein has been shown in biopsies from chloracne, which was not found in 12 controls [15]. Another pathway important in the pathophysiology of chloracne is the transcription factor Nrf2, a key regulator of cellular stress response (ROS detoxification). Effects of Nrf2 on the skin however is controversial; on one side, UVB-induced ROS damage and keratinocyte apoptosis were reduced in transgenic mice expressing Nrf2 in keratinocytes, and on the other side in vivo these mice induced infundibular acanthosis, hyperkeratosis, and cyst formation. These features were linked to upregulation of epigen (a growth factor and novel Nrf2 target, as well as secretory leukocyte peptidase inhibitor (Slpi) and small proline-rich protein 2d (Sprr2d). SLPI, SPRR2, and epigen was also upregulated in dioxin stimulated keratinocytes. In addition, there is a correlation between Nrf2 with increased malignancy and chemoresistance of tumor cells and Nrf2 activation mutations in cutaneous squamous cell carcinomas [16].

In contrast to acne vulgaris, chloracne is very therapy-resistant. Studies showed that systemic retinoids, neither non-steroidal anti-inflammatory drugs nor systemic steroids were not effective to improve severe chloracne. In a case study, however, tumor necrosis factor -α (TNF-α) inhibition in combination with dermabrasion seemed to improve the skin [14].

In 2010, high internal levels of PCBs were discovered in workers of a transformer recycling company in Germany, where PCB-contaminated material was not handled according to proper occupational hygiene procedures [17]. Thereafter ongoing studies as part of a prospective surveillance program were initiated [18]. In an earlier study immunological parameter of the cohort were evaluated. Effects on the proportion of CD19 positive B-cells among lymphocytes and a negative correlation of PCB 114 with serum IgM, and of PCB-28 with suppressor T-cell and NK-cells were found. Higher chlorinated congeners were positively related with CD25 on T-cells. No effects on INF-γ production by T-cells and killing by NK-cells were seen [19]. In addition, cutaneous manifestations such as hyperpigmentation and chloracne were found in the higher PCB- exposed workers, as well as a higher incidence of cutaneous malignancies [20]. Earlier in vitro study showed an altered regulation of several genes involved in immune response and inflammation as well as an upregulation of matrix metalloproteinase such as MMP-2, MMP-7, and MMP-9 in DL-PCB treated peripheral blood mononuclear cells (PBMCs). One of the most dominant upregulated genes in this experiment included the inflammatory mediators CC-Chemokine Ligand 7 (CCL7), CC-Chemokine Ligand 20 (CCL20), C-X-C motif chemokine 2 (CXCL2), Interleukin 1β (IL-1β) and Interleukin 6 (IL-6) [21]. The aim of this current study was to identify whether these in vitro PCB-regulated genes, which play a key factor in inflammatory processes, are upregulated as well in in vivo in skin biopsies of PCB exposed individuals, in order to provide more insight into the pathophysiological effects of PCBs on gene expression level.

## 2. Materials and Methods

### 2.1. Study Group

This study was approved by the Institutional board of the RWTH Aachen University (EK 176/11) and was conducted according to the principles expressed in the Declaration of Helsinki and its amendments. Following verbal consent, written informed consent was obtained from all participants prior to inclusion in the study.

The participants of the current study were part of the HELPcB (Health effects in high level exposure to PCB) surveillance program. In this program, workers of a transformer recycling company exposed to high levels of PCBs were included. Yearly blood samples were obtained in the first three years and then biannually from all participants of this PCB exposed cohort as a part of the surveillance program. Further details are published elsewhere [18]. Of the total cohort, 34 participants with skin lesions were approved for a biopsy after informed consent. Nine participants with lower (background) PCB levels were excluded from examination of gene regulation in the skin.

After local anesthesia with 1% prilocaine (AstraZeneca, London, Great Britain) two 4 mm punch biopsies were taken. One punch biopsy was used for histological examination and one punch biopsy (or surplus skin after nevus excision) of unaffected skin was used for RNA isolation. Tissue samples were stabilized in RNA later (Qiagen, Hilden, Germany), and were mechanically disrupted and homogenized by using a tissue lyser (Qiagen, Hilden, Germany). Total RNA was extracted with Nucleo Spin RNA II (Macherey-Nagel, Düren, Germany), according to the protocol of the manufacturer.

### 2.2. Determination of PCB

Detailed information about determination of PCBs in plasma is described elsewhere [17].

Shortly, we measured serum PCB in the blood using gas chromatography with mass spectrometry (GC-MS) in blood plasma [22]. We measured the non-dioxin-like indicator congeners PCB 28, 52, 101, 138, 153, 180 and calculated their sum (ndl-PCB-sum). For the dioxin-like PCBs, congeners PCB 77, 81, 105, 114, 118, 123, 126, 156, 157, 167, 169 and 189 were measured and their toxic equivalency (TEQ) value was calculated. If only 10% or less than 10% of the measurements did exceed the limit of detection (LOD), the congener was discharged from further investigation. This was the case for PCB 77, 81, 126 and 169. The LOD for every congener was 0.01 µg/L blood plasma. For quality control purposes, bovine serum was spiked with all analytes at a concentration of 0.4 μg/L and included in every analytical series. The relative standard deviation of the between-day precision ranged between 2.4%–8.8%, depending on the congener. Accuracy of our results for the indicator congeners was assured by biannual successful participation in a round robin organized in Germany (www.g-equas.de, 24 January 2020).

For all participants, serum cholesterol and serum triglycerides were measured. As suggested by Bernert et al. (2007), we used the CDC-short formula to calculate total blood lipids (TL) and used TL for lipid standardization of the PCB plasma levels.

Levels of 17 dioxins (7 Polychlorinated dibenzodioxins (PCDDs) and 10 Polychlorinated dibenzofurans (PCDFs)) as well as hexachlorobenzene (HCB) and dichlorodiphenyldichloroethylene (DDE) were measured in the serum of the participants using high resolution gas chromatography mass spectrometry (HR-GC-MS). More details and preliminary results of chromatography with 23 of the participants are published elsewhere [23].

### 2.3. RNA Isolation and qRT-PCR

RNA isolation and RT-qPCR analysis was performed as described elsewhere [24]. TaqMan experiments were carried out on an ABI Prism 7300 sequence detection system (Applied Biosystems, Weiterstadt, Germany) using Assays-on-Demand gene expression products for CCL7 (Hs00171147_m1), CCL20 (Hs01011368_m1), CXCL2 (Hs00601975_m1), IL-6 (Hs00985641_m1), interleukin (IL)-1β (Hs00174097_m1), MMP-9 (Hs00234579_m1), EPGN (Hs02385425_m1), NRF2 (Hs00232352_m1) according to the manufacturer’s recommendations. For all measurements, the exact same amount of nucleic acid material was loaded by each qPCR reaction. All measurements were performed in triplicates in separate reaction wells. CT values were measured using qRT-PCR. Due to the limited amount of sample RNA, no validation with housekeeping genes was possible.

### 2.4. Statistical Analysis

To analyze whether there is a statistically significant correlation between PCBs or PCDD/Fs with cutaneous gene expression, we used SPSS.23 [25]. Because the correlation was mostly not typically linear in the scatter diagram, we choose the Spearman’s correlation coefficient.

As dependent values we used the calculated regulation of MMP9, CCL-7, CCL-20, CXCL2, IL-1β, IL-6, EPGN, NRF2. The levels of serum PCBs (single congeners, sum-ndl- and dl-PCBs) and serum dioxins (PCDD/Fs) were the independent values.

To quantify the relationship of two variables, a Spearman’s rho was used. To adjust for possible confounding factors, the partial correlation coefficient was used. The level of significance was 5% (*p* = 0.05). Sex, age, smoking status and total blood lipids were included as covariates. Q-Q residual plots and row histograms were used to observe the data distribution, skewness was determined. To adjust for multiple testing we conducted a false discovery rate test [26].

For each analysis scatterplots of predictor and criterion were observed to identify outliers with a possibly significant influence on the result. The particular analyses were repeated without the out-layers. Each time the results were consistent after exclusion of possible outliers. Therefore, all presented results include all cases.

## 3. Results

### 3.1. Discriptive Statistics of the Cohort

Descriptive statistics of the cohort can be found in Table 1. We included 25 participants with elevated PCB levels who agreed to a skin biopsy after informed consent. The mean age was 47.98 (29–87) years, mean plasma ndl-PCB levels were 31.81 (2.15–178.4) μg/L, mean plasma dl-PCB levels were 6.12 (0.50–36.68) μg/L, and mean dioxin (PCDD/F) levels were 31.66 (5.55–109.66) TEQ pg/g fat.

### 3.2. Confounding Factors

While age, gender, BMI and serum lipids could be a confounding factor in the model, we analyzed if there was a statistically significant correlation between these endpoints and the measured genes. All the participants were asked for current disease status and current intake of medication.

Statistical evaluation of confounding factors is displayed in Table 2. None of the confounding factors (age, gender, BMI, smoking status, and serum lipids) were correlated to the measured gene expression (see Table 2). Earlier study showed a negative correlation between CYP1A1 and smoking [27].

### 3.3. Regulation of Cutaneous Genes

In this study we measured MMP-9, IL-1β, CCL7, CCL20, CXCL2, IL-6, EPGN and NRF2 in skin biopsies. Possible correlations with levels of dioxins (PCDD/Fs), the sum PCBs, PCB congeners (lipid adjusted) and mixtures in the blood (plasma) were calculated using Spearman’s correlation coefficient. Results are displayed in Table 3.

For MMP-9 we found a statistically significant correlation with PCB 156 (*p*: 0.021, ρ: 0.332) and the sum of the DL-PCB congeners (*p*: 0.027, ρ: 0.506).

An almost significant correlation was found with the sum of the NDL-PCBs (*p*: 0.056, ρ: 0.439).

For IL-1β, we found statistically significant correlations with the sum of the NDL-PCB congeners (*p*: 0.016, ρ: 0.587), PCB 138 (*p*: 0.04, ρ: 0.545), PCB 153 (*p*: 0.032, ρ: 0.560), PCB 180 (*p*: 0.048, ρ: 0.537), PCB 114 (*p*: 0.024, ρ: 0.574), PCB 156 (*p*: 0.048, ρ: 0.533 ), PCB 157 (*p*: 0.040, ρ: 0.542), PCB 167 (*p*: 0.016, ρ: 0.581), the sum of the DL-PCB congeners (*p*: 0.012, ρ: 0.592), the sum of all PCB congeners (lipid adjusted) (*p*: 0.032, ρ: 0.533) and dioxin (PCDD/Fs) (*p*: 0.040, ρ: 0.654). For the expression of CCL7 a correlation with the sum of the NDL-PCB congeners (*p*: 0.014, ρ: 0.332), but not with any other PCB congeners or dioxin levels, were found. Only the sum of the DL-PCBs showed an almost significant correlation with CCL7 (*p*: 0.059, ρ: 0.418).

**Figure 1:**
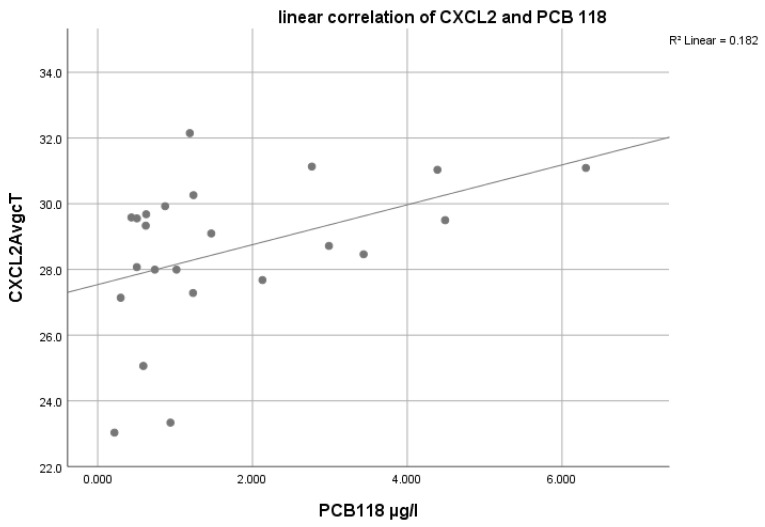
Plasma levels of PCB 118 (in ng/g lipids) and the regulation in CXCL2.

For CCL20 we found a correlation with the PCDD/Fs (*p*: 0.040, ρ: 0.639). An almost significant correlation was found with the sum of the NDL- (*p*: 0.056, ρ: 0.438) and DL-PCBs (*p*: 0.059, ρ: 0.407). For CXCL2 we found statistically significant correlations with the sum of the NDL-PCB congeners (*p*: 0.048, ρ: 0.494), PCB 118 (*p*: 0.044, ρ: 0.506, see Figure 1), the sum of the DL-PCB congeners (*p*: 0.012, ρ: 0.56), and the sum of all PCB congeners (lipid adjusted) (*p*: 0.032, ρ: 0.516).

For IL-6 we found a significant correlation with the sum of the dl-PCB congeners (*p*: 0.032, ρ: 0.478), and PCB 118 (*p*: 0.044, ρ: 0.502).

For HCB and DDE no significant correlation with MMP-9, CCL7, CCL20, CXCL2, IL-1β or IL-6 was found (data not shown).

### 3.4. Regulation of Cutaneous Genes Correlated with Oxidative Stress and the Epidermal Growth Factor (Nrf2 and EPGN)

In addition to the above mentioned genes, we examined whether Nrf2 (Nuclear Factor (Erythroid-derived 2)-like 2 NFE2L2), which regulates the response to oxidative stress, was influenced by PCB blood levels [28]. EPGN as a ligand of the epidermal growth factor receptor (EGFR) was also determined using RT-qPCR. We found no correlation with EPGN or NRF2 (see Table 3). Further statistical analysis showed that EPGN was significantly positively related to CCL7 (*p*: 0.049, ρ: 0.457). As expected, NRF2 was related to MMP9 (*p*: 0.016, ρ: 0.543). Additionally, a statistically significant correlation with CCL7 (*p*: 0.009, ρ: 0.583) and CXCL2 (*p*: 0.049, ρ: 0.489) was found.

## 4. Discussion

### 4.1. Regulation of CCL7, CCL20, CXCL2, IL-1β, MMP-9 and IL-6

We found an upregulation of genes of importance in in immune response and inflammation (CCL7, CCL20, CXCL2, IL-1β, MMP-9 and IL-6) at the mRNA level in skin biopsies of PCB exposed workers.

This is in agreement with in vitro results of earlier study in PBMCs. In this experiment an upregulation of matrix metalloproteinase such as MMP-2, MMP-7, and MMP-9 in DL-PCB treated PBMCs was observed [21]. This is in line with other studies who found that AhR ligands such as 2,3,7,8-tetrachlorodibenzo-p-dioxin (TCDD) upregulate expression of matrix metalloproteinases in vitro. It was hypothesized that induction of extracellular matrix components such as the matrix metalloproteinases may contribute to dioxin-induced cancer invasion and metastasis [29,30].

Additionally, certain chemokines were one of the most prominent upregulated genes in earlier in vitro experiments. RT-qPCR upregulated genes for PCB180 exposed PBMCs included: CCL 1, CCL 20, IL1-α, IL1-β, IL-6. In vivo major targets of dioxin were reported: cytokines (interleukin-1B, TNF (Tumor Necrosis Factor)), growth factors (EGRF, TGF-B (transforming growth factor) and different genes in the apoptotic (Fas ligand, caspases, genes of the Bcl-2 family) and angiogenic pathways (vascular endothelial growth factor (VEGF) and the plasminogen activator cascade, and angiogenin, which also influences proliferation and differentiation pathways in the skin. It is known that DL-PCBs are able to induce an inflammatory response, probably via the AhR pathway [31]. An upregulation of AhRR, the negative feedback regulator of AhR, was found in DL-PCB treated PBMCs. In previous in vivo studies we could not confirm this [21]. However, activation of non-AhR pathways following PCB exposure are also described [32].

**Figure 2:**
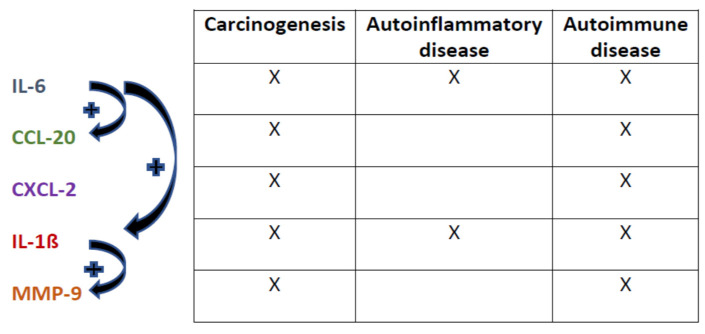
Interaction of IL-1β, CCL-20, CXCL2, MMP-9 and IL-6 and its effects on carcinogenesis, autoinflammatory disease and autoimmune disease [29,30,37].

We observed a significant upregulation of the inflammatory mediators CCL7, CCL20, CXCL2, IL-1β and IL-6 at the mRNA level. Upregulation of these inflammatory mediators has been linked to the pathogenesis of autoimmune disorders, and plays a potential role in tumor progression and metastasis (see Figure 2) [33,34,35] as well as the pathogenesis of autoimmune neurological diseases (CCL20) [34]. Interestingly, levels of IL-1β in PBMCs decreased with decreasing PCB levels of exposed workers in a previous study [27].

We found an upregulation of IL-6 in skin biopsies of PCB exposed workers. In agreement with our study, increased IL-6 levels (together with IFNγ, Il-2, and IFNγ/IL-4 ratios) were found in livers and serum of PCB (Aroclor 1254) treated rats [36]. An upregulation of IL-6 has been reported in several kinds of malignancies, and works as a tumor promotor in the tumor microenvironment through multiple pathways including the NF-kB and MAPK/ERK signaling pathway, including promotion of proliferation, angiogenesis and invasiveness [37]. In addition, elevated IL-6 levels are a sign of poor prognosis in most tumors [38].

While the classical IL-6 signaling pathway decreases the pro-inflammatory cytokines TNF-α and IL-1β, the trans-signaling pathway activates these pro-inflammatory pathways leading to chronical inflammation and tumor promotion [37]. IL-6 is also produced by adipocytes and is thought to be a reason why obese individuals have higher endogenous levels of CRP [39].

It is suggested that PCB induced NF-κβ activation leads to a significant increase in inflammatory agents mRNA and protein expression.

Due to the limited size of the skin biopsies, resulting in insufficient amounts of RNA, it was not possible to confirm dysregulation of more genes which were found in previous in vitro study [21].

Other studies showed that differentially expressed genes were found in a study on human B lymphoblastoid cells exposed to PCB-153. One of the differentially expressed genes, as in agreement with our study, was CCL-20, which was suggested to be a possible biomarker [40]. One mice study showed IL-6 shifts macrophage polarization towards tumor-promoting, CCL-20 producing, macrophages. CCL-20 promotes colitis-associated colorectal cancer by recruiting CC-chemokine-receptor-6 (CCR-6)-expressing B cells and γδ T cells through chemotaxis [41].

Serum IL-1β (together with IL-17, IL-23, and TNFα) was significantly upregulated in PCB exposed Yusho victims (Japanese citizens previously exposed to remarkably high levels of PCB/PCDF) compared to normal population. Here, it was suggested that Yusho victims have dysregulated TH17 cell-mediated immune responses that may be linked to inflammation [42]. One study showed that IL-8 was upregulated in Benzo(a)pyrene (BaP) treated human keratinocytes in a AhR-ROS-dependent regulation, which might explain the exacerbation of certain skin diseases such as psoriasis and acne due to cigarette smoking [43].

In vivo several major targets of TCDD were reported: cytokines (interleukin-1β, TNF (Tumor Necrosis Factor)), growth factors (EGRF, TGF-B (transforming growth factor) and different genes in the apoptotic (Fas ligand, caspases, genes of the Bcl-2 family) and angiogenic pathways (vascular endothelial growth factor (VEGF) and the plasminogen activator cascade), and angiogenin, which also influences proliferation and differentiation pathways in the skin [44].

Only one other (background) human exposure study (a Canadian study on First Nations communities), reported a positive association between Aroclor 1260 (PCB 153, PCB 170, PCB 180 and PCB 187) and cytokine levels (including Il-1β) in the blood [45].

To our knowledge, our study is the first showing an upregulation of MMP-9, CCL7, CCL20, CXCL2 or IL-6 in skin samples of PCB exposed individuals. Due to extensive questionnaires, we were able to identify possible confounding factors (age, smoking status, alcohol consumption, exposure to pesticides, past medical history, medication). The upregulation was found for multiple PCB congeners; however we have to keep in mind that the PCB congeners are correlated to each other as well.

However, due to limited amounts of RNA we were not able to make normalization with endogenous housekeeping genes.

Normalization was performed using the same amounts of RNA in each sample, which is the simple first step to reduce experimental error. In addition each sample was measured in triplicates. However, no control for error introduced at the reverse transcription or PCR stages can be assured with this method [46]. Accurate normalization should be performed by using a set of multiple housekeeping genes in further study. Another limitation of the study is the absence of a control group with healthy non-exposed individuals.

### 4.2. Nrf2 and Epigen

We did not find a direct effect of PCBs on the expression of Nrf2. The role of this protein in the skin however is controversial; on one side, UVB-induced ROS damage and keratinocyte apoptosis were reduced in transgenic mice expressing caNrf2 in keratinocytes, and on the other side in vivo these mice induced infundibular acanthosis, hyperkeratosis, and cyst formation. These features were linked to upregulation of epigen (a growth factor and novel Nrf2 target, as well as secretory leukocyte peptidase inhibitor (Slpi) and small proline-rich protein 2d (Sprr2d). SLPI, SPRR2, and epigen were also upregulated in dioxin stimulated keratinocytes. More worrying is a correlation between Nrf2 with increased malignancy and chemoresistance of tumor cells and Nrf2 activation mutations in cutaneous squamous cell carcinomas [16]. The carcinoprotective and procarcinogenic activity Nrf2 has been outlined in one review study [47]. As in agreement with our study, the correlation between Nrf2 and MMP9 was found and linked to its procarcinogenic activity through promotion of invasion and metastasis.

## 5. Conclusions

In this preliminary study we found an upregulation of immunological markers CCL7, CCL20, CXCL2, IL-1β, IL-6 as well as MMP-9 in vivo in skin samples of PCB exposed individuals with higher PCB levels. Confirmation of this finding should be performed with a large gene-set using for example next generation sequencing. In addition, further epidemiological study of PCB exposed individuals is warranted to see whether a possible upregulation of inflammatory genes can cause a higher incidence of malignancies and inflammatory disease in PCB exposed individuals.

## Figures and Tables

**Table 1:** Descriptive statistics of the cohort.

Descriptive	Age (*n* = 25)	BMI (*n* = 25)	Plasma ndl-PCB Levels (*n* = 25) μg/L	DL-PCB Levels (*n* = 25) µg/L	Dioxin Levels (*n* = 25) pg/g Fat
**Mean (range)**	47.98 (29–87)	28.8 (21.0–40.9)	31.81 (2.15–178.4)	6.12 (0.50–36.68)	31.66 (5.55–109.66)
**Median**	48	28.3	16.99	2.75	23.83

Descriptive statistics for age (y), BMI (kg/m^2^), Plasma ndl-PCB (μg/L), DL-PCB (μg/L) and dioxin levels (pg/g fat) are displayed in Table 1. The smoking status was as follows: 17 out of 25 (68%) were actual smokers, 7 out of 25 (28%) were ex-smokers, only 1 out of 25 (4%) never smoked.

**Table 2:** Confounding factors.

Confounding Factors		MMP-9	IL-1ß	CCL7	CCL20	CXCL2	IL-6	EPGN	NRF2
**Age**	ρ	−0.049	0.067	0.041	0.122	−0.013	−0.055	−0.246	−0.342
	*p*-value	0.815	0.749	0.846	0.562	0.950	0.795	0.310	0.152
**Smoking**	ρ	−0.019	−0.075	0.117	−0.253	0.026	−0.087	0.277	0.253
	*p*-value	0.929	0.720	0.578	0.223	0.900	0.680	0.250	0.297
**Serum lipids**	ρ	0.170	−0.065	0.316	−0.018	0.000	−0.124	−0.202	0.170
	*p*-value	0.417	0.756	0.124	0.930	1.000	0.614	0.408	0.417
**BMI**	ρ	−0.042	−0.218	−0.049	0.038	−0.177	−0.101	−0.099	−0.061
	*p*-value	0.844	0.294	0.815	0.858	0.398	0.632	0.686	0.803

Statistical evaluation of the confounding factors (age, gender, BMI, smoking status, and serum lipids). None were correlated to the measured regulation of gene expression.

**Table 3:** Correlation between dioxin (PCDD/Fs) and plasma PCB levels and cutaneous gene expression.

PCB		MMP-9	IL-1ß	CCL7	CCL20	CXCL2	IL-6	EPGN	NRF2
**PCB28**	ρ	0.289	0.135	0.281	0.339	0.352	0.326	0.267	−0.007
	*p*-value	0.278	0.594	0.278	0.278	0.278	0.278	0.358	0.977
**PCB 52**	ρ	0.173	0.147	0.152	0.010	0.092	0.062	0.181	−0.268
	*p*-value	0.774	0.774	0.774	0.961	0.879	0.879	0.774	0.774
**PCB101**	ρ	0.103	0.109	−0.011	0.130	0.139	0.246	0.227	−0.310
	*p*-value	0.722	0.722	0.958	0.722	0.722	0.722	0.722	0.722
**PCB138**	ρ	0.395	0.545 *	0.265	0.354	0.428	0.327	0.210	−0.102
	*p*-value	0.133	0.04	0.268	0.166	0.132	0.1776	0.445	0.679
**PCB153**	ρ	0.392	0.560 *	0.294	0.346	0.445	0.328	0.252	−0.098
	*p*-value	0.139	0.032	0.205	0.176	0.104	0.176	0.341	0.689
**PCB180**	ρ	0.375	0.537 *	0.275	0.367	0.395	0.247	0.161	−0.095
	*p*-value	0.142	0.048	0.293	0.142	0.142	0.312	0.582	0.700
**sum NDL-PCB**	ρ	0.439	0.587 *	0.332 *	0.438	0.494 *	0.368	0.213	−0.058
	*p*-value	0.056	0.016	0.014	0.056	0.048	0.114	0.435	0.814
**PCB105**	ρ	0.358	0.321	0.314	0.295	0.452	0.505	0.254	−0.032
	*p*-value	0.203	0.203	0.203	0.203	0.092	0.080	0.335	0.898
**PCB114**	ρ	0.362	0.574 *	0.261	0.430	0.455	0.376	0.097	−0.163
	*p*-value	0.120	0.024	0.276	0.085	0.085	0.120	0.691	0.576
**PCB118**	ρ	0.409	0.434	0.379	0.345	0.506 *	0.502 *	0.341	−0.077
	*p*-value	0.084	0.080	0.099	0.121	0.044	0.044	0.175	0.753
**PCB156**	ρ	0.332 *	0.533 *	0.226	0.354	0.355	0.234	0.125	−0.171
	*p*-value	0.021	0.048	0.369	0.210	0.210	0.369	0.611	0.553
**PCB157**	ρ	0.344	0.542 *	0.247	0.398	0.404	0.279	0.069	−0.111
	*p*-value	0.184	0.040	0.312	0.131	0.131	0.282	0.778	0.745
**PCB167**	ρ	0.392	0.581 *	0.264	0.413	0.418	0.314	0.107	−0.118
	*p*-value	0.104	0.016	0.271	0.104	0.104	0.203	0.663	0.663
**PCB189**	ρ	0.321	0.512	0.275	0.336	0.328	0.176	0.161	−0.176
	*p*-value	0.234	0.072	0.293	0.234	0.234	0.510	0.510	0.510
**sum DL-PCBs (TEQ)**	ρ	0.506 *	0.592 *	0.418	0.407	0.564 *	0.478 *	0.274	−0.054
	*p*-value	0.027	0.012	0.059	0.059	0.012	0.032	0.294	0.825
**sum PCBs (lipid adjusted)**	ρ	0.408	0.533 *	0.325	0.348	0.516 *	0.422	0.247	−0.051
	*p*-value	0.086	0.032	0.149	0.141	0.032	0.086	0.351	0.836
**sum PCBs**	ρ	0.422	0.568 *	0.32	0.407	0.488	0.381	0.199	−0.091
	*p*-value	0.088	0.024	0.159	0.088	0.052	0.096	0.473	0.71
**WHO2005_** **Teq_PCDD_F**	ρ	0.446	0.654 *	0.475	0.639 *	0.475	0.432	0.152	0.079
	*p*-value	0.144	0.040	0.144	0.040	0.144	0.1444	0.776	0.829

Correlations were calculated using spearman’s correlation coefficient. All PCB congeners are lipid adjusted (ng/g lipids). Values with sum PCBs are presented in lipid adjusted (ng/g lipids) and wet based values (μg/L). PCDD/Fs were measured in TEQ pg/g fat *: Level of significance *p* < 0.05. PCB 77, 81 and 169 excluded, because <10% above LOD.

## Data Availability

The data presented in this study are available on justified request from the corresponding author. The data are not publicly available due to Data Protection Regulation (DSGVO).
